# Genomic variations in Mycobacterium tuberculosis from the lungs and blood of HIV-infected individuals in Uganda: insights into compartmentalization

**DOI:** 10.4314/ahs.v24i4.2

**Published:** 2024-12

**Authors:** Hellen Nakabuye, Dickson Aruhomukama, Ronald Galiwango, David P Kateete

**Affiliations:** 1 Department of Immunology and Molecular Biology, School of Biomedical Sciences, College of Health Sciences, Makerere University; 2 Department of Medical Microbiology, School of Biomedical Sciences, College of Health Sciences, Makerere University; 3 African Center of Excellence in Bioinformatics and Data-Intensive Sciences, College of Health Sciences, Makerere University

**Keywords:** Mycobacterium tuberculosis, genomic variations, bioinformatics, Uganda

## Abstract

Mycobacterium tuberculosis (MTB) clinical strains are relatively varied at the genome level. This in-silico study analyzed genomic differences between MTB isolates from the blood and lungs of TB-HIV positive cohorts in Uganda. The hypothesis was that isolates from the blood have distinct SNPs and INDELs that make them better survivors. Twenty-four MTB-blood and -lung sequences were aligned against the H37Rv reference genome and analyzed using BWA-MEM, IGV, SAMtools, FreeBayes, and SnpEff. Comparative analysis revealed that MTB-blood isolates had 11 virulence genes with distinctive non-synonymous SNPs involved in increasing colony-forming units, lowering host survival, enhancing tissue pathology, and allowing for human host persistence. The majority of INDELs were found in non-virulence genes, with the remainder in both MTB-blood and -lung sequences. The study suggests that MTB-blood isolates have distinctive SNPs that explain their capacity to persist outside of the lungs. However, further research is needed to understand the significance of these SNPs in the pathogenesis of MTB.

**Impact:**

Mycobacterium tuberculosis (MTB) clinical strains have high genomic variability, and there is a knowledge gap on the genomic differences between MTB isolates from the blood and lungs of TB-HIV positive patients in Uganda. This study found that MTB-blood isolates had 11 virulence genes with distinctive non-synonymous SNPs that may contribute to their capacity to persist outside of the lungs. These findings provide insight into the genomic basis of MTB adaptation in different host environments, but further research is needed to fully understand the significance of these SNPs in MTB pathogenesis.

## Introduction

Tuberculosis (TB) is still a major global public health issue; it is one of the top ten causes of death and the leading cause of death from a single infectious agent (above HIV/AIDS)^1^,^2^. According to World Health Organization (WHO) estimates, 1.4 million people died of TB in 2019 (including 208,000 HIV-positive individuals)^3^,^4^. In 2019, Uganda ranked 16th out of 30 nations with the highest TB burden (responsible for 9.6% of new TB infections), with an estimated 102,000 new cases per year^3^.

Tuberculosis is usually restricted to the lungs in immune-competent individuals (referred to as pulmonary TB (PTB))^5^,^6^. Although MTB bacilli continue to spread beyond the lung parenchyma into the blood (termed extrapulmonary TB (EPTB)), causing disease in the peritoneal cavity, pericardium, meninges, lymph nodes, and intra-abdominal organs in people with immune suppression, such as HIV infection^7^–^9^.

Although the bulk of TB initiatives focus on the prevention and treatment of PTB, approximately 1 in every 5 cases of TB is classified as EPTB, and EPTB accounts for more than half of all TB cases among HIV-positive patients^10^,^11^. Due to a combination of advanced HIV, concurrent opportunistic infections, and late TB diagnosis and treatment, EPTB has been linked to a significant mortality rate in these patients^10^,^11^.

Extrapulmonary TB accounted for 15 to 20% of all TB cases in 2015, according to the WHO^10^,^11^. The annual global incidence of EPTB has been steadily growing over the last decade, according to reports^10^,^11^. Changes in TB control procedures and HIV have both been blamed for the rise in EPTB cases^10^,^11^. In addition, the cure of infectious TB patients may have resulted in an increase in yearly EPTB case identification^10^,^11^.

Strains of MTB, particularly clinical strains, have a wide range of genomic variety, ranging from a few SNPs^12^,^13^ to large-scale genomic rearrangements (INDELS)^12^,^13^. Even though the majority of deletions in clinical cases of active TB are thought to occur in genes that encode non-disease pathogenesis essential proteins, some deletions could theoretically result in a selective advantage at certain stages of infection or even enable escape from the host immune response (survival) or spread^12^. Nine MTB complex (MTBC) lineages associated with humans have been recognized worldwide^14^,^15^. Despite the fact that various lineages are spread differently, certain lineages dominate specific geographic regions and human populations^16^,^17^. The pathophysiology of these lineages varies, according to growing evidence, however this has mostly been demonstrated in animal models^18^–^20^. Nonetheless, their differing effects on human TB remain mainly unknown^16^–^18^. There is no consensus on whether the distribution of MTBC lineages and sub-lineages is due to microbial or host influences^16^–^18^. In recent Ugandan studies, the MTBC Uganda family (L4-U), a sub-lineage of lineage 4, has been linked to the bulk of TB cases^16^,^21^. A region of difference (RD) 724, spoligotype fingerprint^33^–^36^, ^40^, and 43 spacers absent), and several SNPs define the MTBC Uganda family 16,22,23. Recent research has reclassified the L4-U family as MTB sensu stricto, which was formerly classified as Mycobacterium africanum sub-type II, based on advances in molecular characterization^16^.

The genetic variations between sequences of MTB Uganda family isolates from the lungs and blood of TB-HIV positive cohorts in Uganda were assessed in this study, with the hypothesis that sequences of isolates from blood have SNPs and INDELs that promote the isolates' better survival.

## Methods

### Study design

This was an in-silico matched pair case-control study. It was performed between October 2021 and January 2022. The cases in this study were MTB-blood sequences. The controls were MTB-pulmonary sequences. MTB-blood sequences were collected from the LAM evaluation study, which was performed in Uganda in 2011. The MTB-pulmonary sequences were collected from the Community Health and Social Networks of Tuberculosis (COHSONET) trial, which was also performed in Uganda in 2012.

### Selection of samples

FASTQ reads from 12 MTB-blood sequences (cases) and 12 MTB-pulmonary sequences (controls) were examined in this study. These sequences were all of the MTB Uganda family, a sub-lineage of MTB lineage 4. The sequences were all confirmed to belong to this family using three (3) SNPs that identify the MTB Uganda family (L4-U) namely: (Rv0006_0238n, Rv0040c-0619n, and Rv2949c-0375s).

### Bioinformatics analysis

Using fastq-dump (v2.9.6), the 24 sequences were retrieved from the European Nucleotide Archive. These were unzipped, and sequence quality control (QC) was performed using FastQC and/or MultiQC (v0.10.0). The phred score for MTB-blood samples was >25 while that for MTB-pulmonary samples was >18. The sequences were aligned against the H37Rv (Ref Seq: NC_000962.3) reference genome using BWA-MEM (v0.7.17) and the number of threads was set to 20. Integrative genome viewer (IGV) (v1.7.1) was used to check alignment quality, and the alignment file was converted from sam to bam format using SAMtools (v0.1.19) for easy browsing, and then sorted using the same tool^24^. The default settings for SAM tools were used for both converting and sorting. The sorted bam files were indexed, and Free Bayes (v1.3.4)^25^ was used to call (identify) SNPs and INDELs. SnpEff (v4.0)^26^ was used to annotate the SNPs and INDELs. After annotation with SnpEff, the results files were filtered to obtain the Chromosome, Position, Reference, Alternate, and Info columns. The Info column was further filtered to extract variant names, gene names, and gene numbers, which were stored into a text file. The text file was retrieved using the ‘cat’ and ‘grep’ commands to extract variants from different genes. The genes where the blood-specific variants occurred were investigated to determine whether they are known to play a role in the virulence of MTB, and if so, what specific role they played.

## Results

### The sequences and quality control

All of the sequences were almost whole genomes, with at least 4.0 billion base pairs (bp), or 90.9% of the overall genome size. All 12 MTB-blood sequences had a Phred score >25. Their GC content ranged between 64% and 65%. The percentage of duplicates (Dups) ranged from 19.9% to 39.1%. SNPs were the most common variations in these sequences. The Phred score of all 12 MTB-pulmonary sequences was >18. Their GC content was >62%. The percentage of Dups ranged from 4.5% to 28.5%. SNPs were also the most common variations in these sequences.

### SNPs unique to MTB-blood sequences

Patterns were reported for SNPs. Patterns were defined as SNPs that appeared in 5 of the 12 sequences. The majority of the SNP patterns affected 5 sequences in 18 genes, followed by 6 sequences in 4 genes, and then 10 sequences in 3 genes. Eight and 9 sequences showed SNP patterns in >1 gene (2 genes) while 7 sequences affected 1 gene. Only 1 SNP (Rv2823c, 3131473) affected all 12 sequences. These SNP patterns occurred in both non-virulence and virulence genes and ranged from high (2), modifier (3), moderate (14), and low (12) impact SNPs (Table 1).

**Table 1:** List of SNPs unique to MTB-blood sequences

Genes	Position	Impact	SNP Frequency (Number of Sequences)
**sigG**	218599	Modifier	5
**Rv0278c**	334440	Moderate	6
**Rv0278c**	336005	Low	10
**senX3**	579284	Modifier	5
**rpfA**	964798	Low	6
**rpfA**	964822	Low	6
**rpfA**	964966	Low	10
**rpfA**	964984	Low	6
**PE_PGRS22**	1218971	Moderate	7
**lpqY**	1377568	Low	5
**PE_PGRS30**	1864047	Moderate	5
**Rv1907c**	2153813	Moderate	5
**lppF**	2172526	High	5
**katG**	2154724	Moderate	5
**ahpC**	2726338	Moderate	9
**plcC**	2627946	Low	5
**Rv2038c**	2284456	Moderate	5
**PE_PGRS38**	2423334	Moderate	10
**Rv2264c**	2541414	Modifier	8
**Rv2293c**	2564368	High	5
**Rv2823c**	3131473	Moderate	12
**Rv2869c**	3180988	Moderate	5
**fadD26**	3244113	Low	5
**Rv3272**	3653988	Moderate	5
**Rv3273**	3656206	Moderate	5
**PE_PGRS52**	3803433	Moderate	8
**Rv3545c**	3984321	Low	5
**Rv3748**	4197399	Moderate	9
**Rv3785**	4231865	Low	5
**Rv3785**	4231874	Low	5
**fbpA**	4266647	Low	5

### SNPs affecting virulence genes

The SNPs unique to blood sequences affected 11 virulence-related genes, with the bulk of the SNPs having a low impact (5 SNPs), followed by moderate impact (4 SNPs), and modifier impact (2 SNPs). The SNP (Rv2823c, 3131473) occurred in a non-virulence gene (Table 2).

**Table 2:** List of SNPs affecting virulence genes

Genes	Position	Impact	SNP Frequency (Number of Sequences)
**sigG**	218599	Modifier	5
**senX3**	579284	Modifier	5
**lpqY**	1377568	Low	5
**PE_PGRS30**	1864047	Moderate	5
**katG**	2154724	Moderate	5
**ahpC**	2726338	Moderate	9
**plcC**	2627946	Low	5
**Rv2869c**	3180988	Moderate	5
**fadD26**	3244113	Low	5
**Rv3545c**	3984321	Low	5
**fbpA**	4266647	Low	5

Genes and their role in virulence. The majority of the affected virulence gene products increase colony forming units (CFUs) in the lungs and organs of their hosts. The product of the ahpC gene, which had the most common SNP in the virulence genes (common in nine blood sequences), escalates host tissue pathology (Table 3).

**Table 3:** Role of gene products of implicated virulence genes

Genes	Role of gene product	References
**sigG**	Increases CFUs in lungs	(27,28)
**senX3**	Increases CFUs in lungs	(29,30)
**lpqY**	Reduces host's survival	(27,31)
**PE_PGRS30**	Increases host's tissue damage	(32,33)
**katG**	Increases CFUs in lungs	(34,35)
**ahpC**	Increases tissue pathology	(36,37)
**plcC**	Increases CFUs in lungs	(38,39)
**Rv2869c**	Increases CFUs in lungs	(27,40)
**fadD26**	Increases CFUs in lungs	(41,42)
**Rv3545c**	Has unclear affiliated roles in virulence	(43)
**fbpA**	Increases CFUs in organs	(44,45)

## Discussion

We aimed to understand the genomic variations that promote MTB survival in the blood of HIV- infected individuals. This was accomplished by identifying SNPs and INDELs that were specific to MTB-blood sequences, determining the common genes in which the SNPs and INDELS occurred, and the pathways in which the genes were involved to enhance MTB survival in blood. We found that MTB-blood sequences had distinct non-synonymous SNPs. These SNPs were found in multiple virulence genes and possibly explain why MTB survives better in HIV-infected people's blood, but the majority of the INDELs found in MTB-blood sequences were found in non-virulence genes.

The true role of all SNPs mentioned in this study is difficult to ascertain. The MTB genome includes 4 million bp and 3959 genes, with 40% of them having their function characterized and another 44% being speculated to have functional relevance^12^.

In a study by Musser and colleagues^46^ that investigated 24 genes encoding target proteins for the immune response of 16 different MTB isolates, it was found that 19 genes were unaffected, and only 6 nucleotide polymorphism sites were found in the 5 genes that did show change. According to Musser and colleagues, SNPs, are likely to arise approximately once every 10,000 bp (approximately 400 SNPs for the whole genome).

Later, Fraser and colleagues^47^ claimed a higher incidence of polymorphism (approximately 1 in 3000 bp) after extensive comparison studies between H37Rv and CDC1551 strains. They looked at both synonymous and nonsynonymous nucleotide polymorphisms in their study. The need for precise SNP frequency estimation was emphasized in their study. Several other studies appear to support the value of 1 synonymous nucleotide change per 10,000 synonymous sites in structural genes (12,48). Nonetheless, recent studies have cited even a much lower mutation rate (approximately 0.24 to 0.5 SNPs per genome per year)^49^,^50^.

Using genomic analysis, Forrellad and colleagues^27^ identified 14 regions (regions of difference, or RD1–14) in the reference laboratory strain MTB H37Rv, which aided in the discovery of pathogenicity-related chromosomal genes.

Mycobacteria lack classical virulence factors such as toxins, and many of the virulence genes of MTBC species are also conserved in non-pathogenic mycobacteria^15^,^17^,^27^. The bulk of MTB virulence genes encode lipid pathway enzymes^51^, cell surface proteins^52^, regulators^53^, and signal transduction system proteins^54^, as well as another set of genes involved in mycobacterial survival in the hostile environment of host macrophages^27^.

Clinical MTB strains, according to Satta and colleagues^12^, demonstrate a wide spectrum of genomic variability that ranges from a few SNPs to large-scale genomic rearrangements. Some SNPs and INDELs may provide a selective advantage during specific stages of infection or transmission, as well as allowing the MTB to evade the host immune response or become drug resistant^12^. Indeed, mutations in 28 virulence and non-virulence genes were found to be interesting in this study (summarized in table 1). However, only 11 virulence genes from MTB-blood sequences were found to have distinct non-synonymous SNPs (the function of these genes is summarized in Table 3). Overall, these are reported to be involved in pathways that increase CFUs in the lungs and organs, lower host survival, increase host tissue damage, and enhance tissue pathology, allowing for human host persistence. This supports the hypothesis that MTB-blood isolates have distinct SNPs that allow them to survive longer than MTB-pulmonary isolates, allowing these strains to stay in the blood of their hosts.

## Limitations

This study did not demonstrate a selective advantage for the non-synonymous SNPs that were identified as they could also be explained by drift within different tissues.

## Conclusions

MTB-blood isolates had 11 virulence genes with distinctive non-synonymous SNPs, according to the genetic variation of the samples. They may have a better chance of surviving than MTB-pulmonary isolates because of these specific SNPs. SNPs could be a unique property of these isolates, explaining their capacity to enter and persist in blood from the lungs. More research is needed to better understand the role of these SNPs in the pathogenesis of TB, as well as to see if any of the genes involved by the SNPs can be exploited as therapeutic targets for the development of new drugs.

## Data Availability

The datasets generated and/or analyzed during the current study are available in the European Nucleotide Archive. Blood: https://www.ebi.ac.uk/ena/browser/view/PRJEB10577?show=reads (Sample IDs: ERR990532, ERR990534, ERR990536, ERR990540, ERR990556, ERR990554, ERR990552, ERR990550, ERR990548, ERR990546, ERR990544, ERR990542). Lungs: https://www.ebi.ac.uk/ena/browser/view/PRJNA663279?show=reads (Sample IDs: SRR12634532, SRR12634533, SRR12634534, SRR12634535, SRR12634536, SRR12634537, SRR12634538, SRR12634539, SRR12634540, SRR12634541, SRR12634542, SRR12634543)
